# Hydroxycholesterol binds and enhances the anti-viral activities of zebrafish monomeric c-reactive protein isoforms

**DOI:** 10.1371/journal.pone.0201509

**Published:** 2019-01-17

**Authors:** Melissa Bello-Perez, Alberto Falco, Beatriz Novoa, Luis Perez, Julio Coll

**Affiliations:** 1 Instituto de Biología Molecular y Celular, Universidad Miguel Hernández (IBMC-UMH), Elche, Spain; 2 Institute of Marine Research (IIM), CSIC, Vigo, Spain; 3 Department of Biotechnology, Instituto Nacional Investigaciones y Tecnologías Agrarias y Alimentarias, INIA, Madrid, Spain; Emory University, UNITED STATES

## Abstract

C-reactive proteins (CRPs) are among the faster acute-phase inflammation-responses proteins encoded by one gene (*hcrp*) in humans and seven genes (*crp1-7*) in zebrafish (*Danio rerio*) with importance in bacterial and viral infections. In this study, we described novel preferential bindings of 25-hydroxycholesterol (25HOCh) to CRP1-7 compared with other lipids and explored the antiviral effects of both 25HOCh and CRP1-7 against spring viremia carp virus (SVCV) infection in zebrafish. Both *in silico* and *in vitro* results confirmed the antiviral effect of 25HOCh and CRP1-7 interactions, thereby showing that the crosstalk between them differed among the zebrafish isoforms. The presence of oxidized cholesterols in human atherosclerotic plaques amplifies the importance that similar interactions may occur for vascular and/or neurodegenerative diseases during viral infections. In this context, the zebrafish model offers a genetic tool to further investigate these interactions.

## Introduction

Previous studies have shown that, in contrast to a single gene-encoding human c-reactive protein (hCRP) [1], seven genes encode zebrafish (*Danio rerio*) CRP1-7 isoforms [2]. CRP molecules are present from invertebrates to vertebrates. In particular, hCRP is a crucial clinical biomarker for inflammation and most recently has been associated with relevant diseases such as those caused by cardiovascular and neurodegenerative disorders [3–5]. All circulating CRP molecules are planar oligomers of ~ 25 kDa monomers. While hCRP is pentameric (p-hCRP), zebrafish CRP5 crystallizes as trimers [6]. However, it is not yet known whether other CRP1-7 isoforms are trimeric and what are their prevalent physiological conformation(s), although some CRP1-7 isoform-dependent heterogeneous biological properties have been most recently described [6,7].

Planar p-hCRP molecules show opposite lipid-recognition and functional-effector faces [5]. It is well known that the recognition face mainly binds phosphocholine heads exposed at the surface of prokaryotic/eukaryotic membranes in a Ca^++^ [8,9]- and phospholipase A_2_ [10]-dependent manner when generated in damaged tissues [5]. Triggered by CRP-Ca^++^-phosphocholine complexes, the functional-effector face binds C1q to activate the classical complement pathway, immunoglobulin Fc receptors to activate phagocytosis [11,12] and other ligands to activate multiple cellular functions [10]. To accomplish these various functions, hCRP shows at least 4 different conformations [5,13]: **i)** inactive serum-circulating p-hCRP, which is present in low concentrations in healthy humans, increasing 100- to 1000-fold after inflammation; **ii)** pro-inflammatory tissue-associated p-hCRP* [4]; **iii)** pro-inflammatory tissue-associated monomeric hCRP (m-hCRP) with wider ligand capacities which include cholesterol (Ch) [14–16] and **iv)** disulfide-reduced m-hCRP that activates lymphoid and many other cellular types [5,16–18]. Despite the different oligomeric structures of p-hCRP and t-CRP5 [6], their protein hydrophobic profiles, two cysteine residues per monomer, Ca^++^-binding amino acid sequences and location of phosphocholine (PC)-binding pockets are highly conserved [19]. On the other hand, previous transcriptomic studies on *crp1-7* genes have demonstrated differential transcript expression in zebrafish tissues [2], in survivors of viral infection [20] and in mutants defective in adaptive immunity [21]. Additionally, unexpected *crp1-7*/CRP1-7 isoform-dependent anti-viral *in vitro* and *in vivo* activities have been described. In most of the above mentioned situations, *crp2/*CRP2 and *crp5/*CRP5 transcripts/proteins were the most modulated compared with *crp1/*CRP1/ and *crp7*/CRP7. These recent findings revealed novel anti-viral CRP1-7 direct or indirect activities in zebrafish that, to our knowledge, have not been described yet for any CRP, including hCRP. Some of the similar properties mentioned above suggest analogous biological functions for p-hCRP and CRP1-7 [7]; however, whether the CRP1-7 isoforms physiologically exist as different oligomeric structures, conformations and/or become specialized in different ligand-binding or biological functions remains largely unexplored.

Widely used as a general biomarker for bacterial infection and inflammation during decades, circulating p-hCRP has been found recently within atherosclerotic lesions and was proposed as a biomarker for cardiovascular diseases [22]. Additionally, the correlation between infections and cardiovascular heart diseases in humans has been demonstrated not only for bacteria but also for several viral infections [23–26]. Thus, although circulating levels of p-hCRP were initially discovered as increasing from ~10 to >500 mg/l during acute-phase responses to bacterial infections, intermediate concentrations of 10–50 mg/l were detected also during viral infections [27], suggesting possible anti-viral function(s). Nevertheless and despite p-hCRP being one of the most investigated risk biomarker molecules in the human cardiovascular field, and an important component of the anti-bacterial innate response [9], to our knowledge, there is no evidence that p-hCRP or m-hCRP possesses antiviral function. The functional significance of the CRP oligomer-monomer conversion (and *viceversa*?) need to be further clarified to evaluate new chemotherapeutic targets [10,28]. Zebrafish may offer a good genetic model to explore such physiological phenomena.

Using *in silico* and *in vitro* studies, we focused on the lipid-docking/binding, anti-viral activities and oligomeric forms of the zebrafish CRP1-7 isoforms and some of their transcript variants. We found that **i)** Ca^++^-independent docking/binding of CRP1-7 to Ch was higher than that to other lipids, **ii)** HOChs were a preferential target for CRP1-7, **iii)** HOChs enhanced the anti-viral direct or indirect effects by zebrafish CRP1-7 in an isoform-dependent manner, and **iv)** CRP2/CRP5 and numerous CRP5 transcript variants have a stronger tendency to fold as trimers than other CRP-7 molecules.

## Materials and methods

### *In silico* docking predictions between zebrafish CRP1-7 and lipids

AutoDock Vina [29] included in the PyRx program package [30] was used to predict the Gibbs free-energy of docking (ΔG) of 60 × 60 × 60 Å grids surrounding the CRP1-7 molecules. When required for comparison with the experimental data, the output ΔG energies were converted to constant inhibition (Ki) values in molar concentrations (M), using the formula Ki = exp([ΔG × 1000] / [R × T]) (R = 1.98 cal/mol, and T = 298°C)[31]. The predicted structures were visualized in PyRx and/or PyMOL (https://www.pymol.org/).

### Cell culture in EPC cell monolayers

*Epithelioma papulosum cyprinid* (EPC) cells from fathead minnow fish (*Pimephales promelas*) were obtained from the American Type Culture Collection (ATCC, Manassas, VA, USA; code number CRL-2872). EPC cell monolayers were grown at 26°C in a 5% CO_2_ atmosphere in RPMI-1640 Dutch modified cell culture medium supplemented with 20 mM HEPES, 10% fetal bovine serum, FBS (Sigma, St. Louis, USA), 1 mM pyruvate, 2 mM glutamine, 50 μg/ml of gentamicin (Gibco) and 2 μg/ml of fungizone [21].

### Preparation of spring viremia of carp virus (SVCV)

The isolate 56/70 of spring viremia carp virus (SVCV) from carp *C*. *carpio* [32], recently renamed *Carp sprivivirus* [33], was replicated in EPC cell monolayers at 26°C, in the cell culture media described above except for 2% FBS and the absence of the CO_2_ atmosphere. Supernatants from SVCV-infected EPC cell monolayers were clarified by centrifugation at 4000 g for 30 min and kept at -80°C [21].

### Estimation of the effects of methyl-betacyclodextrin (MBCD) on SVCV infectivity

EPC cell monolayers treated for 2 h with different concentrations (0–8 mM) of MBCD were incubated with SVCV for 24 h and then were assayed for fluorescent focus-forming units (ffu) (see later). The results were expressed as the percentage of SVCV infectivity calculated by the following formula: 100 × (ffu treated with MBCD / ffu nontreated with MBCD). To assay for viability, EPC cell monolayers treated with MBCD as above were incubated with 0.5 mg/ml of 3-(4,5-dimethylthiazol-2-yl)-2,5-diphenyltetrazolium bromide (MTT) in a Krebs–Hensleit–HEPES buffer (115 mM NaCl, 5 mM KCl, 1 mM KH_2_PO_4_, 1.2 mM MgSO_4_, 2 mM CaCl_2_, and 25 mM HEPES at pH 7.4) for 3 h, the absorbance at 570 nm was measured, and the percentage of viability was calculated by the following formula: absorbance of MBCD treated cells / absorbance of untreated cells. Means and standard deviations (n = 2) were interpolated and smoothed using the cubic B-spline method in Origin Pro 2017 (Northampton, MA, USA).

### Construction of recombinant pRSET-CRP1-7 for *E*.*coli* expression

The corresponding mRNA sequences of the CRP1-7 proteins [7] were used for the design, construction and expression in *E*.*coli*. All the corresponding synthetic DNA sequences were cloned into the pRSET adding poly-histidine tails (polyH) at their C-terminal ends (GeneArt, Regensburg, Germany). The purified plasmids were then transfected into *E*.*coli* BL21(DE3) and grown at 37°C. The resulting recombinant bacteria from the pRSET-*crp1-7* constructs were induced with IPTG at 25°C. Gradient 4–20% polyacrylamide gel electrophoresis (PAGE) and Western blotting were used to detect CRP1-7 expression.

### Construction of recombinant rCRP1, rCRP2, rCRP5, rCRP7 for insect expression

The mRNA sequences of CRP1, CRP2, CRP5 and CRP7 described previously [7], were used for the design, construction and expression in insect cells (GenScript, Piscataway, NJ, USA). Briefly, target DNA containing the gp67 signal peptide + CRP1-7 + Flag (DYKDDDK) + 6 x polyHis sequences (construct size of ~ 3 Kbp) were synthesized and subcloned into the pFastBac1^TM^ baculovirus transfer vector (Invitrogen). The pFastBac1 recombinants were transfected into DH10 Bac^TM^-competent *E*. *coli* cells and bacmids prepared from selected *E*. *coli* clones. Next, recombinant baculoviruses were generated in *Spodoptera frugiperda* (Sf9) insect cells. For that, Sf9 cells cultured in Grace’s insect media (Gibco BRL) with 10% foetal bovine serum, 3% nonessential amino acids and 20 μg/ml gentamicin at 28°C [34] were cotransfected with bacmids and baculovirus using Cellfectin II. The supernatants containing the recombinant baculoviruses were obtained 72 h posttransfection with titers of ~ 10^7^ pfu/ml.

For rCRP expression and purification, 500 ml of Sf9 cell supernatants were harvested 72 h postinfection and were dialyzed against 50 mM Tris, pH 8.0, 500 mM NaCl. The rCRP-containing medium was incubated with Flag or Ni^++^ columns equilibrated with 50 mM Tris, 500 mM NaCl, 5% glycerol, pH 8.0, eluted with 200 μg/ml of the Flag peptide or 150 mM imidazole, dialyzed against equilibration buffer and kept at -20°C until ready for use. Purified rCRPs were loaded onto 8–20% SDS-polyacrylamide gels (BioRad), electrophoresed, and transferred to nitrocellulose membranes (Schleicher & Schuell) to detect specific tag epitopes. The membranes were blocked with phosphate buffered saline containing 0.05% Tween 20 and 4% skim milk, and then were incubated with anti-poly-H monoclonal antibody MAb (Sigma) for 1 h, followed by incubation with anti-mouse horseradish peroxidase-conjugated immunoglobulins (Sigma) and visualization with diaminobenzidine (DAB). The protein concentrations were determined using the bicinchoninic acid (BCA) method [35] and were confirmed by PAGE with BSA as the standard.

### Production of rabbit antibodies to recognize zebrafish CRP1-7 isoforms

To detect CRP1-7 isoforms in lipid-binding assays and after PAGE by Western blotting, anti-CRP1-7 rabbit antibodies (GenScript, Piscataway, NJ, USA) were raised against 3 of the longest more conserved amino acid stretches such as peptide p1 (^18^SYVKLSPEKPLSLSAFTLC), peptide p2 (^189^DWDTIEYDVTGN) and peptide p3 (^129^RPGGTVLLGQDPDSYVGSFC). All p1, p2 and p3 were located at the CRP1-7 surface, as shown by PyMOL modelling of trimeric CRP5 ± Ca^++^ (4PBP.pdb and 4PBO.pdb, respectively) [6] (data not shown). To reduce assay backgrounds, the anti-peptide antibodies were purified by affinity chromatography against the corresponding synthetic peptides coupled to CNBr-activated Sepharose. Only the affinity-purified anti-p3 antibodies bound purified insect-made rCRP2, rCRP5 and rCRP7 on Western blots under denaturing and nondenaturing conditions and recognized EPC cells transfected with pMCV1.4-*crp2-7* by immunofluorescence (data not shown).

### Binding of CRP1-7 to solid-phase lipids

The binding of CRP1-7 to lipids was assayed in solid-phase 96-well plates (Nunc, Maxisorb) by modifying previously described methods [36]. The wells were coated to dryness with several concentrations of ethanol-dissolved lipids and were kept dried until ready for use. To assay for CRP1-7 binding, the plates were first washed with 0.1 M sodium borate, 1 mM CaCl_2_ buffer, pH 8, and then 0.5 μg/well of rCRPs or 10-fold diluted ssCRP1-7 added in 50 μl of the same buffer and incubated for 60 min. After washing, bound CRP1-7 were detected with rabbit anti-p3 and peroxidase-labelled goat anti-rabbit IgG. Peroxidase was finally assayed with OPD as described previously [37,38]. The resulting data were interpolated and smoothed by the cubic B-spline method using Origin Pro 2017 (Northampton, MA, USA).

### Binding of CRP5 pepscan peptides to solid-phase 25HOCh and docking predictions

A series of 15-mer peptides overlapping 5 amino acids of the CRP5 sequence was chemically synthesized by adding an amino-terminal biotin molecule (Chiron Mimotopes, Victoria, Australia). The synthetic pepscan peptides were diluted in distilled water to 4 mg/ml and were kept frozen until use.

To perform the binding experiments, 2 μg of 25HOCh was dissolved in 50 μl of ethanol and was dried into polystyrene wells of 96-well Nunc Maxisorb plates. After washing the plates with 0.1 M borate buffer pH 8, 1 mM CaCl_2_, pepscan peptides (0.05 μg in 50 μl) were added to each of the wells and were incubated for 60 min. After washing, 1000-fold diluted peroxidase-labelled streptavidin were added and incubated for 30 min. After the last wash, OPD was used to detect the amount of peroxidase as described previously [38].

To perform the *in silico* docking predictions, the best modelled CRP5 pepscan peptide sequences predicted in solution by the Mobyle program http://mobyle.rpbs.univ-paris-diderot.fr/cgi-bin/portal.py#forms::PEP-FOLD [39] were docked to all possible predicted conformations of 25HOCh. All the resulting docking data were interpolated and smoothed using the cubic B-spline method in Origin Pro 2017 (Northampton, MA, USA) and the data that best fitted pepscan binding were selected for representation. Validation of such strategy was confirmed by the high correlation obtained among similarly modeled VHSV G protein pepscan 15-mer peptides and previously published binding data to labeled phosphatidylserine [40] and phosphatidylinositol-bisphosphate [41] (data not shown).

### Preparation of pMCV1.4 plasmids encoding *crp1-7*

Each of the chemically synthesized *crp1-7* and green fluorescent protein (*gfp*) genes was subcloned into the pMCV1.4 plasmid as described previously [7]. The resulting pMCV1.4-*crp1-7* and pMCV1.4-*gfp* plasmid constructs were used to transform *E*.*coli* DH5alpha, amplified and isolated using the Endofree Plasmid Midi purification Kit (Qiagen, Germany). Purified plasmid solutions containing 80–100% of plasmid DNA, as shown by agarose gel electrophoresis were stored at -20°C.

### Preparation of CRP1-7-enriched supernatants

To produce ml amounts of CRP1-7-enriched supernatants (ssCRP1-7), 60% confluent EPC cell monolayers in 25 cm^2^ bottles in 5 ml of cell culture medium were transfected with 5 μg of each of the pMCV1.4-*crp1-7* plasmids complexed with 15 μl of FuGENE HD (Promega, Madison, WI, USA) for 24 h at 22° C (transfection efficiency of 15.2–30.4%, n = 3 as estimated by transfection with pMCV1.4-*gfp*). After washing with fresh cell culture medium, the ssCRP1-7 were harvested 3-days later, the cell debris was eliminated by centrifugation, and the supernatants were sterilized by filtration through 0.2 μ filters and stored in aliquots at -80°C until ready for use [7].

### SVCV infection of preincubated EPC cell monolayers with 25HOCh and ssCRP1-7

To detect the effects of 25HOCh and ssCRP1-7 (25HOCh + ssCRP1-7) on SVCV infection, the concentrations of 25HOCh, and ssCRP1-7, as well as the multiplicity of infection (m.o.i.) of SVCV were first optimized (data not shown). Optimal conditions were obtained when the EPC cell monolayers were pre-incubated with 100 μl of 4-fold diluted ssCRP1-7 or ssGFP in RPMI with 2% FBS in the absence or presence of 10 μM 25HOCh for 20 h at 26°C, the monolayers were washed twice, and SVCV was added at 10^−2^ m.o.i. To estimate the extent of SVCV infection, the monolayers were incubated with SVCV for 2 h, washed, and incubated for 24 h at 26°C. The number of infected EPC cells was estimated by flow cytometry after staining with monoclonal anti-SVCV (BioX Diagnostics SA, Jemelle, Belgium) and fluorescein-labelled goat anti-mouse immunoglobulins as described previously [7]. The number of EPC infected cells varied from 29.6–39.7% or 9.9–20.1% (n = 3) after preincubation of the EPC cell monolayers with either 25HOCh or ssCRP1-7 alone, respectively. The results of preincubation with 25HOCh + ssCRP1-7 were expressed as relative percentages of infection ± 25HOCh calculated by the following formula, 100 × (percentage of infected EPC cells preincubated with 25HOCh + CRP1-7 / percentage of infected EPC cells preincubated in absence of 25HOCh and presence of CRP1-7).

### *In silico* modeling of CRP1-7 tridimensional structures

To explore the CRP1-7 tridimensional structures, their protein sequences were automatically modelled (RMSD<0.3 Ă) using the SWISS-MODEL homology server (https://swissmodel.expasy.org/interactive) [42–44]. The tridimensional structures of the target CRP1-7 sequences were predicted after pairwise comparison of the interfaces between the target and best template selected by the program. For each possible interface with > 10 residue-residue interactions, the QscoreOligomer score was calculated and averaged from all predicted interfaces [42,45]. The templates that resulted selected by automatic modeling corresponded to zebrafish CRP5 ± Ca++ (4PBP.pdb and 4PBO.pdb) [6] (RCSB data bank at http://www.rcsb.org/pdb/home/home.do).

## Results

### Preferential docking predictions of zebrafish CRP1-7 to Ch

**Fig 1:**
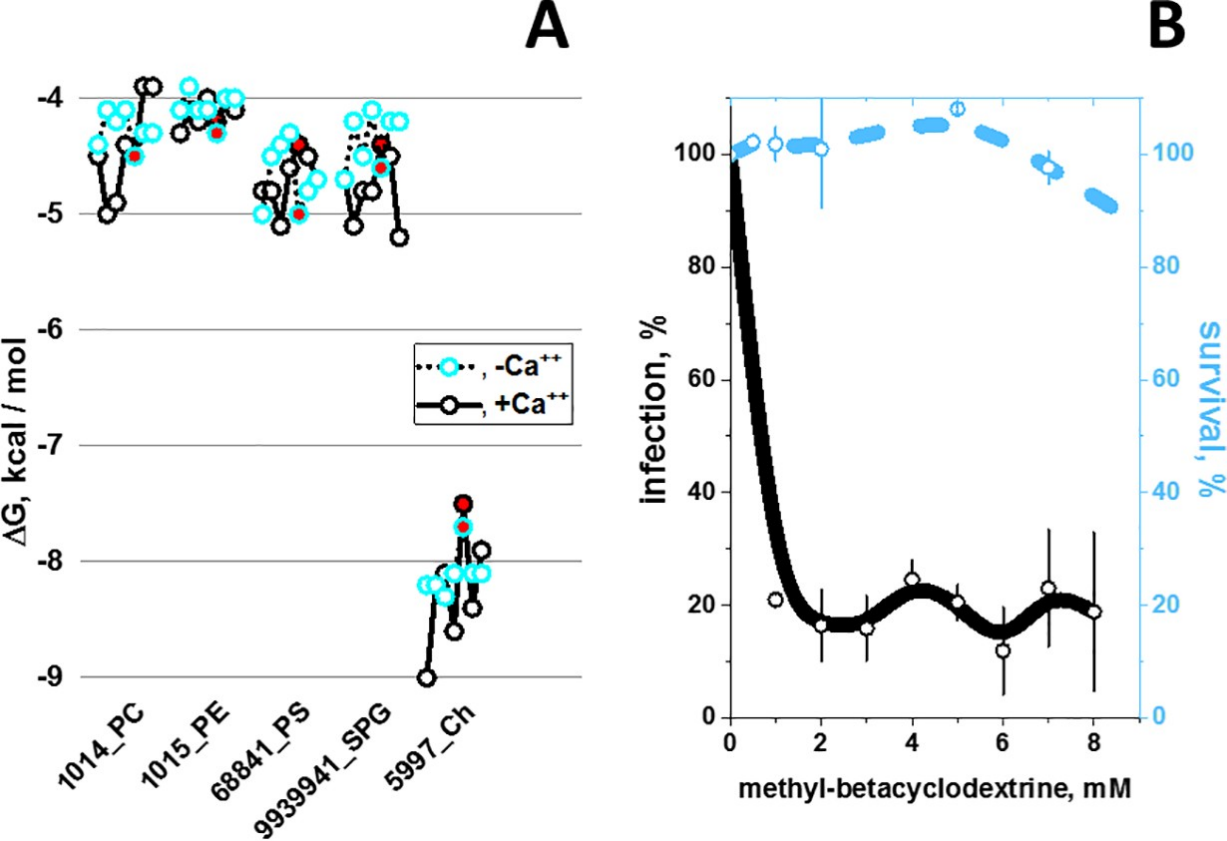
**CRP1-7 preferential docking to Ch (A) and inhibition of SVCV infectivity by methyl-betacyclodextrin (MBCD) (B). A)** Docking predictions to selected lipid-heads and Ch. CRP1-7 were SWISS-modeled using as templates CRP5 (GenBank accession number JF772178.1), 4PBP.pdb (**+Ca**^**++**^) and 4PBO.pdb (**-Ca**^**++**^) 3D-structures [6]. The structures were extracted from *.sdf from PubChem (https://pubchem.ncbi.nlm.nih.gov/search/search.cgi) and converted to *.pdbqt using the Babel program from the PyRx package [30]. **PC**, phosphocholine. **PE**, phosphoethanolamine. **PS**, phosphoserine. **SPG**, palmitoyl sphingomyelin. **Ch,** cholesterol. **Numbers before the names**_, PubMed ID numbers. **Blue open circles**, consecutive CRP1-7 isoforms from left to right in the absence of Ca^++^. **Black open circles**, consecutive CRP1-7 isoforms from left to right in the presence of Ca^++^. **Red circles**, CRP5. **Black lines**, +Ca^++^. **Dot lines**, -Ca^++^. **B)** Effect of methyl-betacyclodextrin (MBCD) on SVCV infectivity. MBCD-treated EPC cell monolayers were incubated with SVCV for 24 h and were assayed for ffu. The results were expressed as infectivity percentages calculated by the following formula, 100 × (ffu treated with MBCD / ffu nontreated with MBCD). To assay for viability, MBCD-treated EPC cell monolayers were incubated with MTT for 3 h, the absorbance at 570 nm was measured and the percentage of viability calculated using the following formula: absorbance of treated cells / absorbance of untreated cells. **Open blue or black circles and their vertical lines**, means and standard deviations (n = 2), respectively. The data were then interpolated and smoothed using the cubic B-spline method in Origin Pro 2017 (Northampton, MA, USA). **Black line,** SVCV infectivity. **Blue dashed line**, EPC cell monolayer viability.

To predict their docking ΔG energies to CRP1-7, the phosphocholine head (PC), other phospholipid heads [46–48] and cholesterol (Ch) [16] were selected because of their hCRP ligand properties. Interestingly, the results predicted the lowest ΔG (stronger binding) for Ch (ΔG ranges from -7.5 to -9 Kcal/mol) compared with phospholipid-heads (ΔG ranges from -4 to -5.5 Kcal/mol). The addition of a glycerol molecule to the phospholipid-heads did not change their predicted ΔG (S1 Table). The results also predicted that Ch docking energies were more Ca^++^-independent than most other lipid-heads (Fig 1A) and predicted alternative docking locations for Ch and other lipid-heads (data not shown). These results were in contrast to the traditionally described phosphatidylcholine-binding preferences of hCRP [46–49]. Thus, although the Ch-binding properties of hCRP were described previously, their stronger binding energies were not [16]. Similar Ch-binding preferences were obtained by docking predictions made in parallel for hCRP and CRP1-7 (S1 Table).

### Membrane Ch sequestration by methyl-betacyclodextrin reduces SVCV infection

To explore whether Ch could be implicated in SVCV infections, EPC cell monolayers were pretreated with methyl-betacyclodextrin (MBCD), a sequestering agent for membrane Ch [50]. Treatment with MBCD from 0.5 to 8 mM lowered the SVCV infectivity to ~ 20% (Fig 1B, black line), while those concentrations exerted no significative effects on cell survival (Fig 1B, blue dashed line). These results confirmed that the presence of Ch in the cell membranes was required for SVCV infectivity. Similar anti-viral activities of MBCD have been described, for instance, for poliovirus [51], pseudorabies [52], hepatitis [53], Sendai [54] and influenza [50,55] viruses. Therefore, these results suggest that the Ch-CRP1-7 interaction may interfere with SVCV infectivity.

Because Ch-containing lipid rafts participate in interactions with hCRP [56], Ch is a key molecule involved in coronary diseases and Ch-related physiological compounds are highly diverse, an screening for other physiological Ch-related compounds was performed before studying any possible interactions among CRP1-7, Ch and viral infections.

### Preferential predicted docking of zebrafish rCRPs to hydroxycholesterols (HOChs)

**Fig 2:**
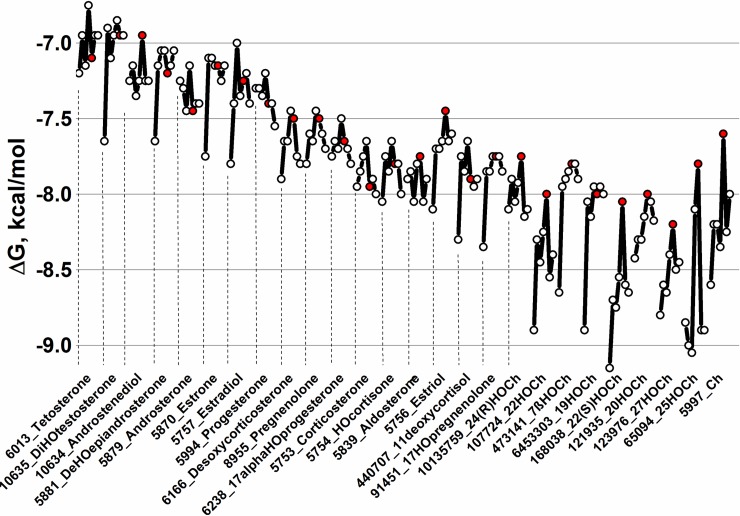
CRP1-7 docking predictions to several Ch-related physiological compounds. CRP1-7 models, Ch-related physiological molecules and ΔG predictions were obtained as described in the legend of Fig 1A. Because the predicted ΔG values in the absence or presence of Ca^++^ were similar (S2 Table), only the mean ΔGs ± Ca^++^ were represented. **Open circles**, consecutive CRP1-7 isoforms from left to right. **Red circles**, CRP5. **Numbers before the names**_, PubMed ID numbers. **HO**, hydroxy. **Ch,** cholesterol.

When 26 Ch-related physiological compounds were docked *in silico* to the modeled tridimensional structures of CRP1-7, stronger binding predictions (ΔG ranges between -7.5 to -9.3 Kcal/mol) were obtained for most of the hydroxy derivatives studied for CRP1-7 (Fig 2 and S2 Table). The ΔG values obtained in the absence or in the presence of Ca^++^ were not significantly different (S2 Table). Most of the lowest ΔG values were obtained for CRP1, while CRP5 showed ~ 0.5–1 Kcal/mol higher ΔG than CRP1, depending on the Ch-related molecule. The most relevant results of these Ch-related docking predictions could be summarized as follows: **i)** water-soluble hydroxy Ch derivatives (HOChs) interacted with CRP1-7 within the lower ΔG ranges from -8.0 to -9 Kcal/mol; **ii)** among the HOChs, most of the lower ΔG values corresponded to CRP1, while most of the highest ΔG values corresponded to CRP5; and **iii)** 25-hydroxycholesterol (25HOCh) was unique among all the studied HOChs because of their lowest ΔG values (~ -9 Kcal/mol). No previous reports on 25HOCh-CRP interactions could be found in the literature.

To explore the existence of other possible Ch-related compounds with still lower ΔG values that could be used as anti-inflammatory chemotherapeutic drugs, a library of 1093 Ch-related synthetic molecules was docked to modeled CRP1-7 tridimensional structures. The frequency distribution of the predicted ΔGs showed a distribution with a mean ± 3 standard deviations = -12 Kcal/mol (S3 Table). Twenty-one Ch-related nonphysiological or synthetic compounds showed the lowest ΔG values from -13.3 to -12 Kcal/mol (Table 1). Most of the new molecules identified contained deuterium, fluorine, bromine or chlorine atoms and 66.6% contained at least one hydroxy group per molecule. Therefore, some of these newly identified Ch-related derivatives could be further employed for drug applications and/or mechanistic studies in the future. Next we tried to confirm some of the docking predictions mentioned above by solid-phase binding assays.

**Table 1:** Ch-related nonphysiological compounds with the best docking predictions to CRP1-7.

ID	Name	CRP	ΔG, Kcal/mol
71749935	**M** Progesterone-**d**3 Glucuronide	CRP5	-13.3
70626502	25-**F**-1α-**HO**Ch	CRP1	-13.3
71749934	**M** Progesterone Glucuronide	CRP5	-13.3
70626891	di**F**-methyl-dodecahydro-cyclopentaphenanthrene	CRP1	-13.0
493972	**F**-11-**HO**-Methyl-DioxoPregnadien-Acetate	CRP5	-12.6
192154	tri**F**lumedroxone Acetate	CRP5	-12.6
240767	**F**metholone 17 Acetate	CRP5	-12.5
95574	**F**-16a,17-(isopropylidenedioxy) Corticosterone	CRP5	-12.5
71748935	20-**HO**Ch-**d**7	CRP5	-12.4
21122966	6-**HO-M** Progesterone 17-Acetate	CRP5	-12.3
102276261	3-[(2-**B**-ethyl)Carbamoyl]Ch	CRP5	-12.3
71749110	**HO-M** Progesterone 17-Acetate	CRP5	-12.2
71748841	4–7 **HO**cholestenone-**d**7	CRP1	-12.1
57357615	17-(Acetyloxy)-**C**-(**C**-methyl)Pregnadienedione	CRP5	-12.1
126456352	24-**HO**Ch-**d**4	CRP1	-12.1
71748930	4-**HO**Ch-**d**7 4-Acetate	CRP5	-12.1
71315435	Cortexone **M**-**d**9	CRP1	-12.1
10476437	Flugestone 17-Acetate	CRP5	-12
71315435	Cortexone **M**-**d**9	CRP5	-12
71315434	Cortexone **M**-**d**8	CRP1	-12
71315434	Cortexone **M**-**d**8	CRP5	-12

Ch-related nonphysiological compound structures were retrieved from several libraries obtained from PubChem in a *.sdf format. To construct the library, 550 Chs, 314 colestens, 73 corticosterones, 41 dehydroepiandrosterones (DHEAs), 107 estriols, 99 pregnenolones, 196 progesterones and 107 HOChs were retrieved. Duplicated and extremely long molecules were eliminated from the total of 1487 *.sdf, resulting in a downsized library of 1093 *.pdbqt archives. After docking, the frequency distribution of ΔG showed two peaks with means at -11 and -7 Kcal/mol, respectively (S3 Table). Only Ch-related compounds with ΔG < -12 Kcal/mol (mean + 3 standard deviations) are shown. **ID,** PubMed number**. HO,** hydroxy**. d,** deuterium**. F,** fluoro-**. C,** chloro-**. B,** bromo. **M**, 17-acetyl-6,10,13-trimethyl-3-oxo-1,2,6,7,8,9,11,12,14,15,16,17-dodeca **HO**cyclopenta[a]phenanthren-16-yl) acetate (medroxy).

### Binding of zebrafish rCRPs to hydroxycholesterols (HOChs), Ch and PC

**Fig 3:**
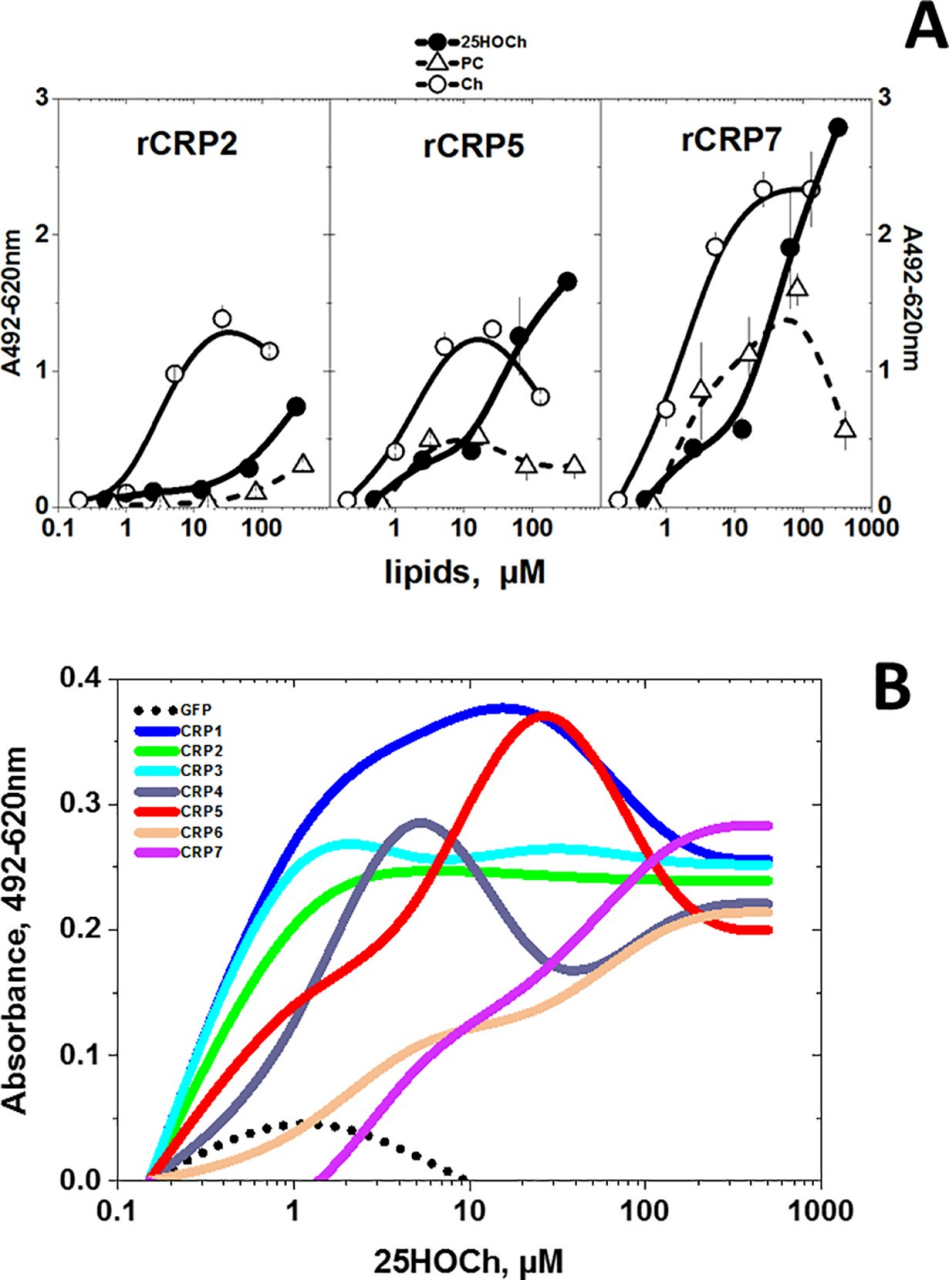
**rCRP (A) and ssCRP1-7 (B) binding to solid-phase lipids.** The binding of purified rCRPs and ssCRP1-7 to selected lipids was assayed by using 96-well plates coated to dryness with several lipid concentrations dissolved in ethanol. The lipid-coated plates were washed and were incubated with rCRP2 or ssCRP1-7 in borate buffer for 1 h in a 50 μl volume. To detect bound rCRP or ssCRP1-7, rabbit anti-CRP p3 peptide, peroxidase-labeled goat anti-rabbit IgG and OPD were used as described previously [37,38]. The means and standard deviation from 2 independent experiments were represented. **A)** rCRP at 0.5 μg/well in borate buffer. **Open triangles**, solid-phase phosphatidylcholine (PC). **Open circles**, solid-phase Ch. **Black circles**, solid-phase 25HOCh. **B)** ssCRP1-7 were 10-fold diluted in borate buffer. Results from one experiment out of three were interpolated and smoothed using the cubic B-spline method in Origin Pro 2017 (Northampton, MA, USA) (see data in S4 Table). **Black points**, supernatant from pMCV1.4-*gfp* transfected cells. **Blue line**, supernatant from pMCV1.4-*crp1* transfected cells. **Green line**, supernatant from pMCV1.4-*crp2* transfected cells. **Light-blue line**, supernatant from pMCV1.4-*crp3* transfected cells. **Gray line**, supernatant from pMCV1.4-*crp4* transfected cells. **Red line,** supernatant from pMCV1.4-*crp5* transfected cells. **Orange line**, supernatant from pMCV1.4-*crp6* transfected cells. **Purple line**, supernatant from pMCV1.4-*crp7* transfected cells.

Because of the recently described anti-viral activities of 25HOCh [57,58] and its highest predicted docking to CRP1-7, its binding was compared with Ch/PC (the former because it is the traditional ligand for hCRP). For the binding assays, we used polystyrene wells coated with the lipids [36]. Using 25HOCh to coat the solid-phase, the binding results confirmed the higher docking of rCRP5/rCRP7 to Ch/25HOCh than to PC (Fig 3A). The binding of rCRP7 to Ch/25HOCh was slightly higher than to rCRP2 or rCRP5 (Fig 3, rCRP7) whereas rCRP2/rCRP5 binding to Ch or PC were relatively low (Fig 3, rCRP2 and rCRP5). To complete the study, we explored all isoforms for binding to 25HOCh using supernatants from EPC cells transfected with pMCV.4-*crp1-7* (ssCRP1-7) as a source for CRP1-7. The results of these experiments showed different concentration-dependent profiles for different CRP1-7, with CRP1 being the most active at the lower 25HOCh concentrations assayed (<10 μM) (Fig 3B and S4 Table) confirming the docking predictions. On the other hand, although CRP7 showed slightly higher binding at >100 μM 25HOCh, similar values were obtained for all ssCRP1-7 at those higher concentrations. The binding of ssCRP1-7 to solid-phase 25HOCh showed relatively lower values than that to rCRPs, most probably due to the lower CRP concentrations in the ssCRP1-7 (compare the ordinate values of Fig 3A and 3B).

### Mapping of both binding and docking energies of CRP5 to 25HOCh

To further clarify 25HOCh binding to CRP1-7, we performed a pepscan approximation to map the interaction. Because m-hCRP, but not p-hCRP is the conformation that preferentially binds Ch [16,17,59], some nonconformational motifs may conserve Ch-/25HOCh-binding activity and thus a pepscan may be used to map at least some conformation-independent binding. Therefore, we selected a pepscan to explore for possible non-conformational interactions of CRP5 with 25HOCh using both solid-phase binding assays and docking predictions.

**Fig 4:**
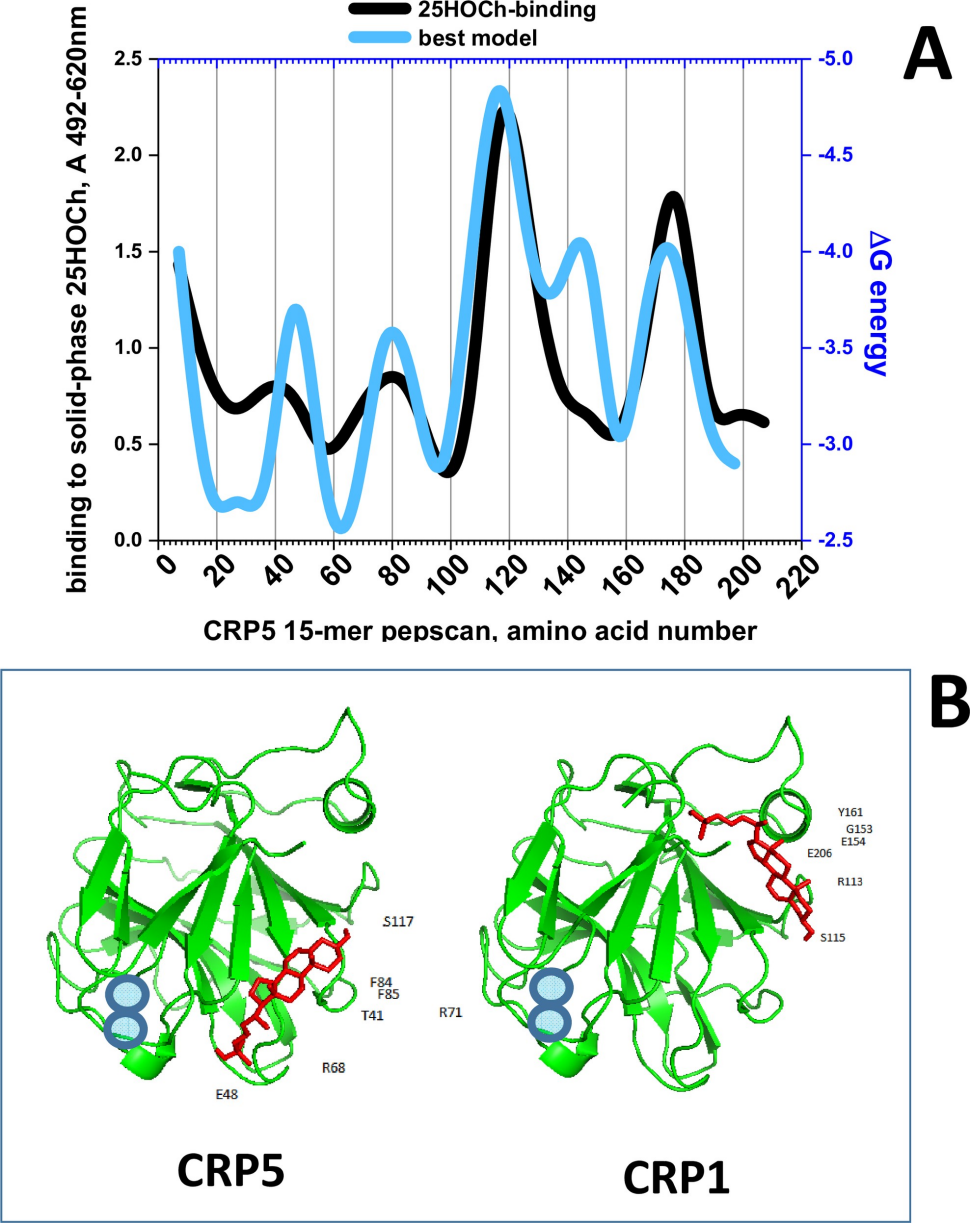
**Solid-phase binding and docking predictions to 25HOCh of CRP5 pepscan peptides (A) and predicted best docking location (B). A)** For the peptide-binding assays, a series of 15-mer peptides overlapping 5 amino acids from CRP5 was chemically synthesized by adding an amino-terminal biotin molecule. Solid-phases were coated with 2 μg per well of 25HOCh into polystyrene 96-well plates. The binding of 0.05 μg of biotinylated pepscan peptides, detection with peroxidase-labeled streptavidin and staining with OPD were then performed. The means from 3 independent experiments were represented and standard deviations omitted for clarity (S5 Table). For the *in silico* docking predictions, the modeled pepscan peptides with the lowest energies were docked to several possible conformations of 25HOCh as described in the methods section. The docking energies that best fitted the binding data were then represented (S5 Table). **Black line**, peptide binding to 25HOCh. **Blue line**, predicted ΔG energy of peptide docking to 25HOCh. **B)** PyMOL representation of the lowest energy structures of CRP5 and CRP1 complexed to 25HOCh (the remaining CRP1-7 were similar). **Green**, CRP amino acid chains. **Red**, 25HOCh. **Blue circles**, Ca^++^ atoms located at the PC-binding pocket [6].

For the peptide binding assays, each of the synthetically biotinylated 15-mer peptides derived from the CRP5 amino acid sequence was incubated with 25HOCh-coated solid-phases. The results showed maximal binding peaks at the ~ 30–50, 70–90, 110–150 and 170–190 amino acid positions (Fig 4A, black line). Similar peaks docked with minimal ΔG to 25HOCh (Fig 4A, blue line). Of the 25HOCh binding/docking peaks identified, only the 30–50 was in a similar region to that of the 35–47 peptide previously identified in hCRP as the main Ch-binding domain [16]. To locate the predicted interaction of 25HOCh with the CRP1-7 tridimensional structures we used PyMol. The 25HOCh docked at the CRP5 interface side with ΔG between -7.5 and -8.4 Kcal/mol (some of the contact positions at T41, E48, R71, F84, F85, S117) (Fig 4A, CRP5). By contrast, the 25HOCh docked at other CRP1-7 effector faces under the α-helix with ΔG between -8.6 and -9.1 Kcal/mol (some of the contact positions for CRP1 at R113, S115, G153, E154, Y161, and E206) (Fig 4B, CRP1). Similar ΔG values (S5 Table) and docking locations were predicted for m- or t-CRP5. Similar docking locations were predicted for 25HOCh and Ch for most CRP1-7 within ± ΔG > ~0.5 Kcal /mol (S5 Table).

Therefore, both the pepscan binding and docking predictions, confirmed the existence of an interaction between 25HOCh and CRP5, which most likely can be extended to all CRP1-7.

### *In vitro* anti-SVCV effects caused by CRP1-7 in the presence of 25HOCh

Hydroxylated Chs (HOChs) are Ch oxidized derivatives with diverse biological activities, most of them correlating with inflammatory responses [60] similar to CRP. Among the HOChs, 25HOCh showed minimal ΔG docking predictions for CRP1-7 (-8 to -9 Kcal/mol, corresponding to concentrations between 1.35 and 0.35 μM) (Fig 3). Among their biological activities, 25HOCh has been related to viral infections [58,61], including the reduction of spring viremia carp virus (SVCV) infection in zebrafish [57]. Because of the Ch-dependence of SVCV infection (Fig 1B), the reduction of SVCV infection by zebrafish ssCRP1-7 [7] was chosen as an example of possible CRP1-7- HOChs interactions affecting the same biological function.

**Fig 5:**
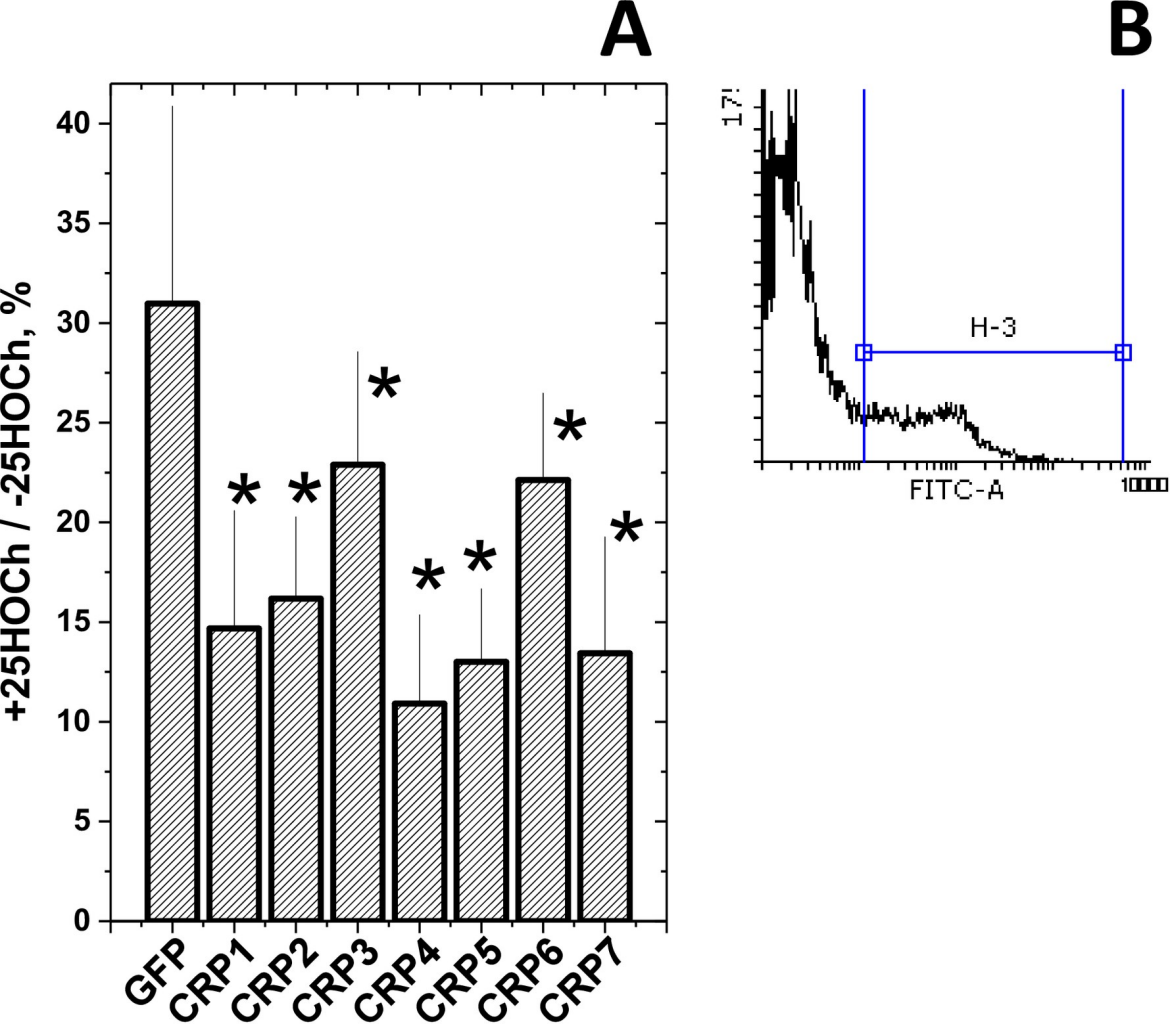
Anti-SVCV infectivity after treatment of EPC cell monolayers with 25HOCh and CRP1-7. **A)** EPC cell monolayers were incubated with 100 μl of ssGFP or ssCRP1-7 4-fold diluted in RPMI with 2% FBS ± 10 μM 25HOCh for 20 h at 26°C. After washing, 10^−2^ m.o.i of SVCV were added and incubated for 24 h. After staining with anti-SVCV and fluorescein-labeled goat anti-mouse immunoglobulins [7], the number of fluorescent cells were estimated by flow cytometry. **B)** Representative aspects of histograms from nonfluorescent and fluorescent cells. The number of SVCV-infected EPC cells varied from 12.7 to 50.6% (n = 5), depending on the experiment. The results were expressed as relative infection percentages calculated by the following formula, 100 × (number of infected cells+25HOCh / number of infected cells -25HOCh). The means and standard deviations of a representative experiment were represented (n = 3). *, statistically < than cells transfected with ssGFP at p < 0.05 (Student's t-test).

Because both 25HOCh [57] and CRP1-7 [7] have demonstrated their independent anti-SVCV activities, their concentrations were first independently titrated at different multiplicities of infection (m.o.i.) of SVCV to maximize the limits of detection when they were to be used together. Under those optimal conditions, the extent of SVCV infections obtained using ssCRP1-7 + 25HOCh (ssCRP1-7 + 25HOCh/ssCRP1-7 ratios) compared with 25HOCh (GFP + 25HOCh) were further reduced by 1.5 to 3-fold depending on the CRP1-7 isoform (Fig 5A). Similar results were obtained with rCRP5 and rCRP7 but not with rCRP2 (data not shown). The above results suggested that 25HOCh in the presence of CRP1-7 further enhanced the anti-viral effects caused by either 25HOCh or CRP1-7 alone. It is still too early to know the mechanisms implicated, because the interaction of 25HOCh with the L polymerase of SVCV [57], or inhibition of glycosylation by 25HOCh in other rhabdoviruses [62], may suggest that the binding of 25HOCh to some viral proteins cannot be excluded. Furthermore, other possible interactions among 25HOCh, CRP (direct effect) and/or other CRP-induced molecules (indirect effects) present in ssCRP1-7, may still explain the above mentioned antiviral effects.

To further explore any possible correlations among CRP1-7 tridimensional structures and 25HOCh binding or antiviral effects, we next studied whether different oligomeric forms were present in ssCRP1-7.

### Insect-made rCRPs suggest their different oligomerization states

The *E*.*coli*-made zebrafish c-reactive protein CRP5 isoform (rCRP5) was crystallized as trimers (t-CRP5), as shown by X-ray studies [6]. However, it is not yet known whether trimers are the physiological form for the remaining CRP1-7 isoforms.

Our first attempts to characterize CRP1-7 isoforms included expression in *E*.*coli*. However, numerous experiments met with irreproducibility, expression failure, high CRP denaturation or low yields, despite the reduction of autoinduction and temperature, and/or recloning of the best-producing clones (data not shown). Most probably some of those results could be explained by the toxicity of the rCRPs to *E*.*coli*.

Alternatively, we explored the production of rCRP1/rCRP2/rCRP5/CRP7 in insect cells.The results showed that while insect-made rCRP2/rCRP5/rCRP7 could be expressed and purified by nondenaturing affinity chromatography, all attempts to purify rCRP1 were unsuccessful. Western blot analysis using anti-polyH antibodies indicated that although small amounts of rCRP1 were present, they were not retained by the affinity columns (data not shown), most likely due to polyH tail inaccessibility, perhaps because of a different conformation of rCRP1 compared with that of the other rCRPs.

**Fig 6:**
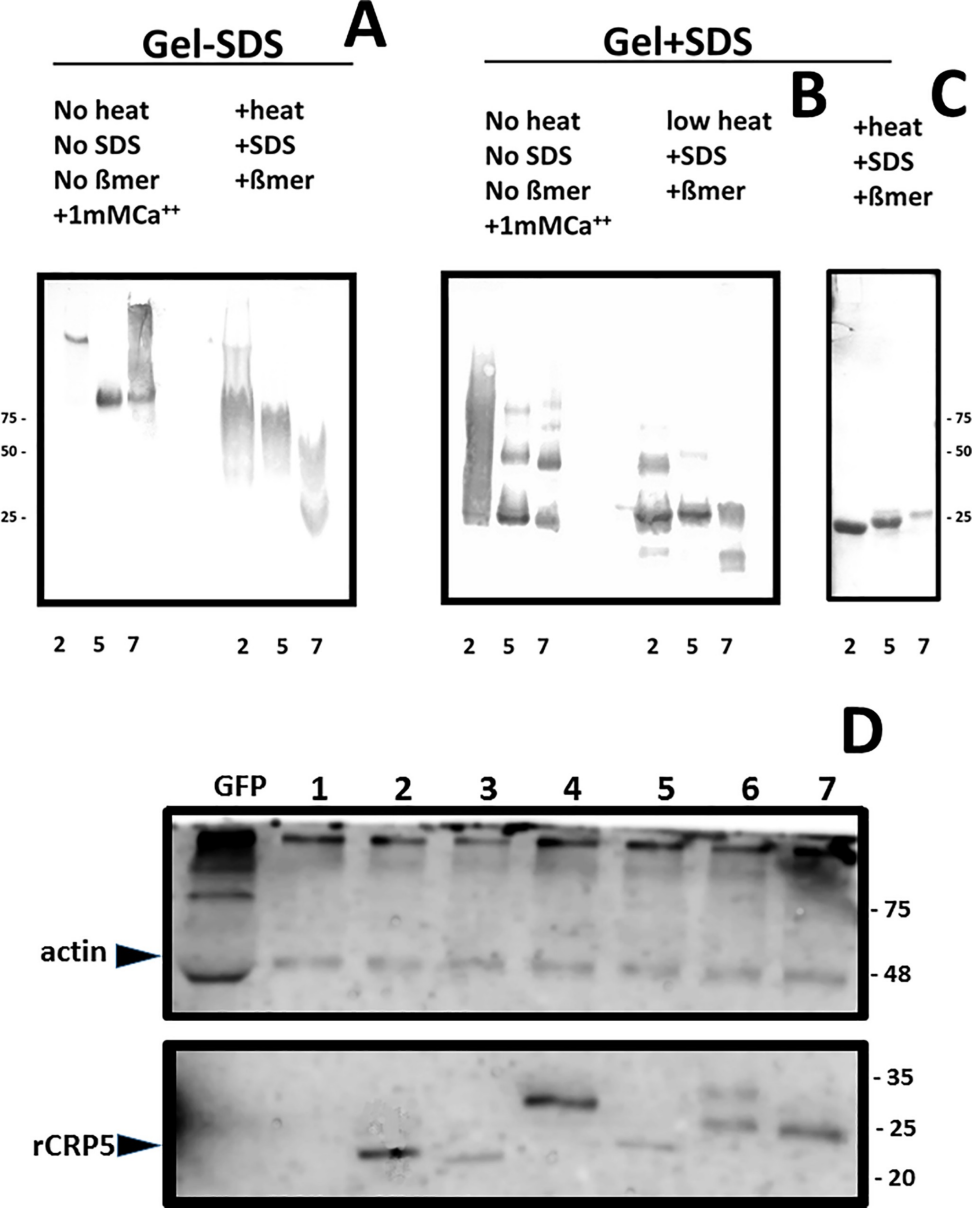
**Polyacrylamide gel electrophoresis and Western blotting of rCRPs (A,B,C) and ssCRP1-7 (D).** The insect-made affinity purified samples were electrophoresed in 4–20% gradient polyacrylamide gels. **A)** Samples of rCRPs prepared and electrophoresed in the absence of SDS in the buffers and stained with Coomassie (nondenaturing conditions). **B)** Samples of rCRPs heated at 100°C in the presence of ß-mercaptoethanol and SDS, electrophoresed in the presence of SDS in the buffers (denaturing conditions) and stained with Coomassie. **C)** Western blotting of the gel B transferred to nitrocellulose membranes, stained with anti-p3 antibody, peroxidase-labeled anti-rabbit and overexposed to diaminobenzidine (DAB) [37]. The ssCRP1-7 were electrophoresed in 15% polyacrylamide gels. **D)** Samples of ssCRP1-7 treated at 100°C in the presence of ß-mercaptoethanol and SDS, electrophoresed in the presence of SDS in the buffers, transferred to nitrocellulose membranes, stained with anti-actin (up) or anti-p3 (down) antibody, peroxidase-labeled anti-rabbit IgG and developed by chemiluminescence [7]. Similar results were obtained with samples electrophoresed under nondenaturing conditions (data not shown). **Numbers around the gels,** molecular weight markers in kDa. **Up left arrow**, position recognized by anti-actin antibodies. **Down left arrow**, position of purified rCRP5 recognized by anti-p3 antibodies. The results are representative of at least 3 experiments.

Polyacrylamide gel electrophoresis (PAGE) in the absence of SDS in the buffers, treating the samples under nondenaturing conditions (no heat, no SDS, no ß-mercaptoethanol and 1 mM CaCl_2_), and Western blotting with anti-p3 antibodies, showed that rCRP2 (calculated isoelectric point IP of 6.35) banded at an apparent molecular weight > 100 kDa, while rCRP5 (IP 4.6) and rCRP7 (IP 4.6) banded at ~ 75 kDa (Fig 6A left). A brief (2 min) treatment of the rCRP samples under denaturing conditions, increased the migration of all rCRP, especially that of rCRP7 (Fig 6A right). Although, in the absence of SDS, the estimation of the molecular weights is not accurate, the results suggested larger sizes for rCRP2/rCRP5 than for rCRP7, according to previous electrophoretic data described for p-hCRP and m-hCRP [63].

By contrast, by applying PAGE in the presence of SDS in the buffers, samples under nondenaturing conditions and Western blotting, all the rCRP displayed similar bands that could be interpreted as residual amounts of trimers (~75 kDa), dimers (~50 kDa) and monomers (~25 kDa) (Fig 6B, left). The number of monomers increased when the samples were briefly treated (2 min) under denaturing conditions; especially for rCRP7, only monomers were detected (Fig 6B, middle). All rCRP became homogeneously monomeric (~25 kDa) when the samples were treated for longer (5 min) under denaturing conditions (Fig 6B, right). The slightly different positions of the monomeric forms could be due to differences in their glycosylation, although posttranscriptional deimidation has also been described in cod CRPs to cause electrophoretic heterogeneity [64].

The most likely explanation for all the above commented data suggest that, while insect-made rCRP2/rCRP5 may exist as an equilibrium among trimers, dimers and monomers, rCRP7 has a stronger tendency to form monomers.

### Only monomeric CRP1-7 could be detected from enriched supernatants

Western blotting of ssCRP1-7 using anti-p3 antibodies, only detected CRP2-7 monomers of ~ 25 kDa with slightly different positions for each ssCRP2-7, with similar profiles under denaturing (Fig 6C, down), 20-fold lower SDS concentration (not shown)[65] and nondenaturing (data not shown) sample and buffer conditions. Similar CRP2-7 levels were present in ssCRP2-7 as shown using actin as an internal control marker (Fig 6C, up). In these experiments, it was not possible to detect the presence of any CRP1 band, most likely because of its lower concentration, because previous results have demonstrated its presence by dot-blot analysis when using concentrated ssCRP1 [7]. Therefore, most likely, all ssCRP1-7 were secreted from EPC transfected cells mainly as monomers. Tridimensional structure predictions were used to further explore these possibilities.

### *In silico* predictions of CRP1-7 tridimensional structures

**Fig 7:**
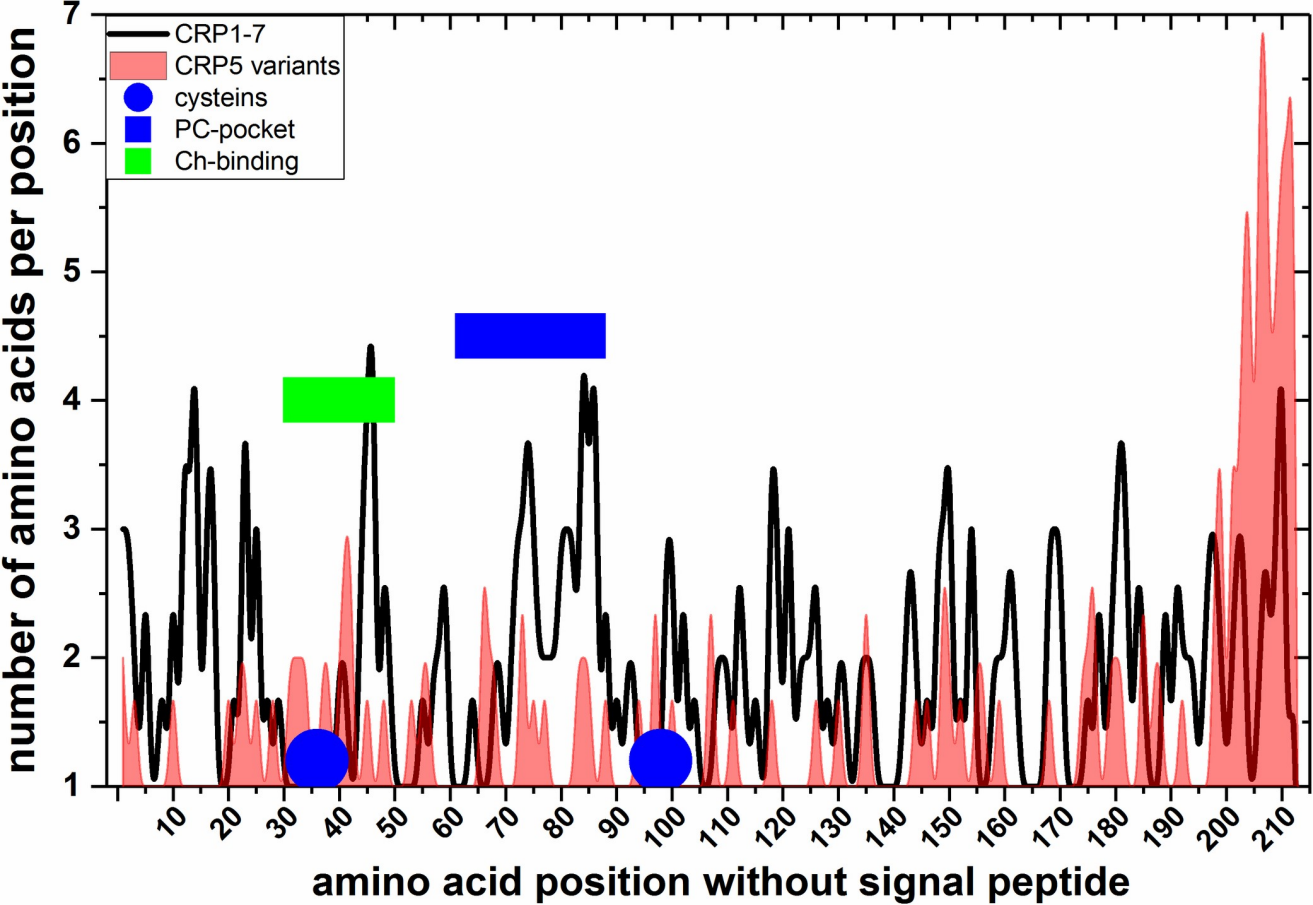
Alignment among EST-derived amino acid sequences of CRP5-transcript variants. Transcript variants corresponding to the zebrafish *crp5* gene were retrieved from 73 ESTs obtained from different zebrafish tissues (UniGene Dr.124528-Dr.162306) [6]. The corresponding ORFs > 100 amino acids translated by the Virtual Ribosome software (http://www.cbs.dtu.dk/services/VirtualRibosome/) were numbered without the signal peptide (^1^FKNL…in CRP5) and were aligned to CRP5 (BC121777). The number of different amino acids per position was represented. The raw data (S6 Table) were smoothed using the cubic B-spline method (Origin Pro 2017, Northampton, MA, USA). **Blue circles**, cysteines. **Blue rectangle**, PC-binding pocket of hCRP. **Green rectangles**, Ch-binding residues of hCRP [16]. **Black line**, number of amino acids per position of CRP1-7. **Red profile**, number of amino acids per position of CRP5 transcript variants.

To obtain more data on the possible tridimensional structures of CRP1-7, their amino acid sequences were modeled using the SWISS-MODEL web program. Automatic modeling showed that only CRP2/CRP5 rendered trimers, while the remaining of the CRP1-7 only modeled as monomers (Table 2). These results could be explained because CRP2/CRP5 have differences in most of their modeling parameters, specially in their torsion-angle potentials, compared with the other CRP1-7 (Table 2). Because the existence of EST from zebrafish in the UniGene Bank classified as CRP5 transcript variants [6] offered another opportunity to test the reliability of the trimer/monomer predictions mentioned above, we explored these sequences by automatic modeling. The corresponding modeling results predicted that 97.8% of the 47 CRP5 longest variant sequences modelled as trimers such as CRP2/CRP5. The comparison of the CRP5 variant amino acid sequences demonstrated 2-3-times more variations downstream of position 200 than in the rest of the molecule (Fig 7, red). On the other hand, most amino acid variations among the CRP1-7 isoforms were in the PC-binding pockets or hCRP-homologous Ch-binding domain (Fig 7, blue or green rectangles, respectively). Therefore, these results predicted the tendency of CRP2, CRP5 and CRP5-transcript variants to oligomerize as trimers and prompted further studies about the biological significance of both CRP isoforms and variants.

**Table 2:** Parameter values of *in silico-*predicted CRP1-7 oligomeric structures.

isoform	Acc.number	Automatic SWISS prediction	QMEAN	Cß	AA	SO	TO
**CRP1**	XM_693995.4	monomer	-0.55	-1.36	**-1.03**	-1.03	0.01
**CRP2**	BC097160	homotrimer	**0.77**	-1.64	**-1.08**	**-0.92**	**1.19**
**CRP3**	BC154042	monomer	0.01	-1.15	-1.15	-1.03	0.51
**CRP4**	BC115188	monomer	-1.81	-1.65	-1.95	-1.33	-1.07
**CRP5**	BC121777	homotrimer	**1.42**	**-0.71**	**-0.89**	**-0.60**	**1.62**
**CRP5-**	Dr.124528-	97.8%	**1.12**	**-0.90**	**-0.99**	**-0.68**	**1.30**
**47 variants**	Dr.162306	homotrimers	±0.31	±0.5	±0.2	±0.4	±0.5
**CRP6**	BC162745	monomer	-0.45	-1.20	-1.17	-1.34	0.15
**CRP7**	BC150371	monomer	-0.42	-1.04	-1.22	-1.28	0.15

The CRP1-7 amino acid sequences [2] were modeled as tridimensional structures using the SWISS-MODEL server with automatic template selection. Additionally, 47 full-length CRP5 amino acid sequences were modeled from 73 zebrafish *crp5* EST variants (UniGene Dr.124528-Dr.162306) [6]. **QMEAN**, estimation of the total similarity to the template, comprising 4 individual Z-score parameters (Cß, all-atom, solvation and torsion). The individual Z-scores compare the predicted tridimensional structures with the template as follows: **i)** Cß atoms of three consecutive amino acids (**Cß**), **ii)** all-atoms (**AA**), **iii)** solvation burial status of the residues (**SO**) and **iv)** torsion angle potentials (**TO**). Low QMEAN score values indicate low similarity to the template. High QMEAN score values indicate high similarity to the template. **Bold**, highest and/or lowest score values. **Gray**, CRP2/CRP5. The mean ± standard deviation (n = 47) of the calculated score values of the CRP5-transcript variants were represented.

## Discussion

The PAGE/Western data and *in silico* predictions, together with the results of 25HOCh binding and enhancement of anti-SVCV effects by ssCRP1-7, may implicate more m-CRP1-7 rather than t-CRP1-7 in those biological functions. However, CRP1-7 may also physiologically exist as an equilibrium of trimers, dimers and monomers, as shown in the cases of CRP2/CRP5 and, to a lower extent CRP7. On the other hand, because m-hCRP can also be produced during *in vitro* manipulations, for instance, by treatments in the absence of Ca^++^ with urea, low-pH or low-salt buffers [65,66], the m-CRP1-7 detected in this work may have been produced by other *in vitro* manipulations (e.g., purification by affinity chromatography in the absence of Ca^++^ or transfection of EPC cells). We may also speculate that t-CRP1-7 or at least CRP2/CRP5 could preferentially exist in fish until an unknown stimulus triggers their conversion to m-CRP1-7 and/or *viceversa*. Similarly, circulating hCRP is pentameric (p-hCRP) [13] and converts to the monomeric form (m-hCRP) after interaction with any exposed phosphocholine heads and/or Ch-enriched lipid rafts of cellular membranes in damaged tissues [16,17,59,63]. It is tempting to speculate that t-CRP2/CRP5 may be functionally analogous to the circulating p-hCRP and m-CRP1-7 could be analogous to the converted m-hCRP. Alternatively, all zebrafish m-CRP1-7 may be synthesized *de novo* as monomers. We may also think of the possibility of heterologous CRP1-7 oligomers. However, any of these possibilities remains speculative until specific reagents could be developed to differentiate each of those isoforms.

Together, the above commented evidence shows that the oligomeric state of CRP1-7 isoforms fine tunes their lipid binding and, at least, some of their resulting heterogeneity of biological functionalities, as suggested previously [2,6,7]. Thus, previous transcriptomic studies on zebrafish *crp1-7* genes have demonstrated differential transcript expression throughout tissues [67], in survivors of VHSV infection [20] and in *rag1*^−/−^ mutants defective in adaptive immunity [21]. Additionally, unexpected isoform-dependent *in vitro* and *in vivo* anti-viral activities were recently described for zebrafish CRP1-7 [7], while similar activities have never been reported for hCRP, or for any other CRP. In all those studies, *crp2/*CRP2 and *crp5/*CRP5 transcripts/proteins were the most modulated during either bacterial and viral infections, correlating with the higher trimeric propensity of CRP2/CRP5 and in sharp contrast to *crp1/*CRP1 and *crp7*/CRP7 which had remained mostly unmodulated. These findings together with the preference of CRP1-7 for hydroxycholesterol derivatives shown in this work, revealed fish primitive anti-viral functional CRP1-7 diversity that may also be relevant to the single-gene-encoded hCRP.

The relevance of these explorations in the CRP1-7 lipid interactions with viral infection diseases may have important implications for human diseases. For instance, the abundance of oxidized Chs in human atherosclerotic plaques amplifies the impact that hCRP-Ch interactions may have for vascular diseases and neurodegenerative disorders during viral infections [58,68,69].

## Supporting information

S1 Table Docking predictions to selected lipid-heads and Ch. The CRP1-7 were SWISS-modeled using the 3D structures CRP5 (GenBank accession number JF772178.1), 4PBP.pdb (**+Ca**^**++**^) and 4PBO.pdb (**-Ca**^**++**^) as templates. The structures of the lipid heads and cholesterol were extracted from *.sdf from PubChem (https://pubchem.ncbi.nlm.nih.gov/search/search.cgi) and converted to *.pdbqt using the Babel program from the PyRx package. Dockings were performed with a grid of 50x50x50 Angstrom. **Yellow background**, data used to derive Fig 1A.
(XLSX)

S2 Table CRP1-7 docking predictions to several Ch-related physiological molecules in the absence and presence of Ca^++^. CRP1-7 models, Ch-related physiological molecules and ΔG predictions were obtained as described in the legend of Fig 2. **Numbers before the names**_, PubMed ID numbers. **HO**, hydroxy. **Ch,** cholesterol.
(XLSX)

S3 Table Docking predictions of binding of Ch-related nonphysiological compounds to CRP1-7. Ch-related nonphysiological compound structures were retrieved from several libraries obtained from PubChem in a *.sdf format. To construct the library, 550 Chs, 314 colestens, 73 corticosterones, 41 dehydroepiandrosterones (DHEAs), 107 estriols, 99 pregnenolones, 196 progesterones and 107 HOChs were retrieved. Duplicated and extremely long molecules were eliminated from a total of 1487 *.sdf, resulting in a downsized library of 1093 *.pdbqt archives. The docking were performed to CRP1-7 modelled in the absence or in the presence of Ca^++^ (crp ±Ca++). **A)** Table of Ch-related compounds ordered from the lowest to the highest ΔG (free-binding energies) in Kcal/mol after docking to CRP1-7. **Yellow background**, data used to derive Table 1. **B)** Distribution of ΔG in relative frequencies. **Black** a**rrow**, cut-off ΔG value chosen to derive Table 1. **C)** Correlation between the ΔGs from the dockings using CRP +Ca^++^ and CRP-Ca^++^.
(XLSX)

S4 Table ssCRP1-7 binding to solid-phase 25HOCh. The binding of ssCRP1-7 to 25HOCh was assayed using plates of 96-wells coated to dryness with 0.15 to 500 μM 25HOCh dissolved in ethanol. The 25HOCh-coated plates were washed with borate buffer and incubated with ssCRP1-7 in borate buffer for 1 h in a 50 μl volume. Bound ssCRP1-7 were detected using rabbit anti-CRP p3 peptide, peroxidase labeled goat anti-rabbit IgG and OPD. Raw absorbances were measured at 492–620 nm. Absorbance obtained with empty wells were subtracted to all data. **Yellow background**, data used to derive Fig 3B.
(XLSX)

S5 Table Solid-phase binding and docking prediction raw data with their calculations of 25HOCh and the CRP5 pepscan interactions. For the 25HOCh-binding, a series of 15-mer peptides overlapping 5 amino acids from the CRP5 sequence were chemically synthesized adding an amino-terminal biotin molecule. Solid-phases were coated with 2 μg per well of 25HOCh into polystyrene 96-well plates. Binding of 0.05 μg biotinylated pepscan peptides, detection with peroxidase-labelled streptavidin and staining with OPD were then performed. For the *in silico* docking predictions, the modeled pepscan peptides with the lowest ΔG energies in solution were docked to all possible conformations of 25HOCh. **n**° **peptide,** position of the middle amino acid of each 15-mer peptide of the pepscan. **1,2,3,4. . . .,** number of replicas of 25HOCh-binding or predicted 25HOCh-CRP5 conformations of 25HOCh in the 25HOCh-CRP5 complexes**. ±sd,** standard deviations**. Poses**, list of ΔG of the predicted complexes for the different conformations of 25HOCh when docked to the CRP5 peptides. **docking best pose**, the pose which resulted in the best fitting to the 25HOCh-binding data. **Bold gray background,** 25HOCh-binding data which was represented in Fig 4A which was represented in Fig 4A**. Bold yellow background**, predicted Kcal/mol ΔG of peptide docking to 25HOCh which best fitted the binding data. *, non-significant highest ΔG energies > -1.1 were adjusted to -2.5 Kcal/mol for best fitting the binding data.
(XLSX)

S6 Table Number of amino acids per position after alignement among EST-derived amino acid sequences of CRP5 and CRP5 transcript variants. Transcript variants corresponding to the zebrafish *crp5* gene were retrieved from UniGene Dr.124528-Dr.162306. ORFs > 100 amino acids were translated by the Virtual Ribosome software (http://www.cbs.dtu.dk/services/VirtualRibosome/), numbered without their signal peptides (^1^FKNL…in CRP5) and aligned to the sequence of CRP5 (BC121777). **Amino acid**, amino acids written in the three or single letter code. **Number**, different amino acids per position in CRP5 and CRP5 EST-derived variants.
(XLSX)

## Abbreviations

BSA: bovine serum albumin
CRP: C-reactive protein
Ig: immunoglobulin
kDa: kilo Daltons
MW: molecular weight
PAGE: polyacrylamide gel electrophoresis
PBS: phosphate-buffered saline
pfu: plaque forming units
SDS: sodium dodecyl sulfate
SVCV: spring viremia carp virus
VHSV: Viral haemorrhagic septicemia virus
EPC: *Epithelioma papulosum cyprinid* cell line
